# Propofol or Thiopental sodium in patients undergoing reproductive assisted technologies: Differences in hemodynamic recovery and outcome of oocyte retrieval: A randomized clinical trial

**Published:** 2014-01

**Authors:** Mohammad Hossein Jarahzadeh, Reza Jouya, Fatemeh Sadat Mousavi, Mohammad Dehghan-tezerjani, Shekoofa Behdad, Hamid Reza Soltani

**Affiliations:** 1 *Department of Anesthesiology and Critical Care, Research and Clinical Center for Infertility, Shahid Sadoughi University of Medical Sciences, Yazd, Iran.*; 2 *Shahid Sadoughi University of Medical Sciences, Yazd, Iran.*; 3 *Department of Anesthesiology and Critical Care, Shahid Sadoughi University of Medical Sciences, Yazd, Iran.*; 4 *Scientific Society of Medicine, Yazd Branch, Islamic Azad University, Yazd, Iran.*

**Keywords:** *Thiopental sodium*, *Propofol*, *Assisted reproductive technology*.

## Abstract

**Background:** Thiopental sodium and Propofol are two widely-used drugs in the induction of anesthesia in assisted reproductive technology (ART). However, the side effects and outcome of recovery from anesthesia of these drugs on ART have not been identified yet.

**Objective: **This study aimed at investigating the side effects and hemodynamic effects of using thiopental sodium and propofal as well as effects of these drugs on pregnancy outcome in ART cycles.

**Materials and Methods:** In this double blinded) randomized controlled trial, 90 woman candidate for ART were randomly divided into two groups. 47 patients received Propofol (2.5 mg/kg) and 43 patients received thiopental (5mg/kg) for anesthesia induction. The entry hemodynamic parameters of the patients were documented. During the anesthesia process, hemodynamic parameters were checked at five-minute intervals.

**Results:** The results of the study showed a statistically significant difference between two groups in terms of their response to verbal stimulation (p<0.001), the normalization time of the rate and quality of breathing (p<0.001), nausea (p<0.001), and vomiting (p<0.001). Also, in comparison with the other group, all these parameters were better in Propofol group. There was found no significant difference between two groups in terms of other variables.

**Conclusion:** Based on the findings of the study, Propofol has fewer known side effects. Vomiting and nausea as two known side effect of anesthesia are significantly lower in patients receiving Propofol than patients who received thiopental.

Registration ID in IRCT: IRCT201303135393N2

This article extracted from M.D. thesis. (Reza Jouya)

## Introduction

Transnvaginal ultrasonography-guided oocyte retrieval is a temporary outpatient procedure requiring a quickly and effectively anesthetic technique with minimal side effects (1). Since the first time anesthetics were found in the follicular fluid controversial theories have been proposed for the deleterious effects of anesthetics on oocyte retrieval during in vitro fertilization (IVF) (2, 3). Some studies suggest that these drugs may adversely affect oocyte fertilization and embryonic development (4, 5). As a result, the optimal anesthetic technique for these assisted reproductive technology (ART) procedures is unknown. Propofol (2, 6 disopropylphenol) is a popular anesthetic drug that is a hypnotic agent. It produces mild to moderate sedation with reported side effects like bradycardia and Asystole (1.4 per 100.000 patients) (4, 5). 

In 1998, Tatone *et al* questioned Propofol’s safety in ART while before that several evidences had confirmed its safety in IVF (6-9). From then on, several studies have been conducted with the aim of evaluating and comparing the efficacy of Propofol and its side effects on oocyte retrieval. This study was conducted to evaluate the side effects, hemodynamic effects, and pregnancy outcome of these drugs in women who were candidate for ART and were suffering from male factor infertility.

## Materials and methods

The double blinded randomized controlled trial method was used in this study. Patients and data collectors were blinded during study. This study was done in Research and Clinical Center for Infertility, Shahid Sadoughi University of Medical Sciences from June to September 2012.

**Samples**

Intra-cytoplasmic sperm injection (ICSI) candidate women at their pregnancy age (15-35 years) were randomly (using even and odd list) and consequently selected during a convenience sampling for the study (47 patients received Propofol versus 43 patients that received thiopental). The criteria based on which the candidates were selected were the lack of infertility problems in the women and the presence of fertility problems in their husbands. 

**Figure 1:**
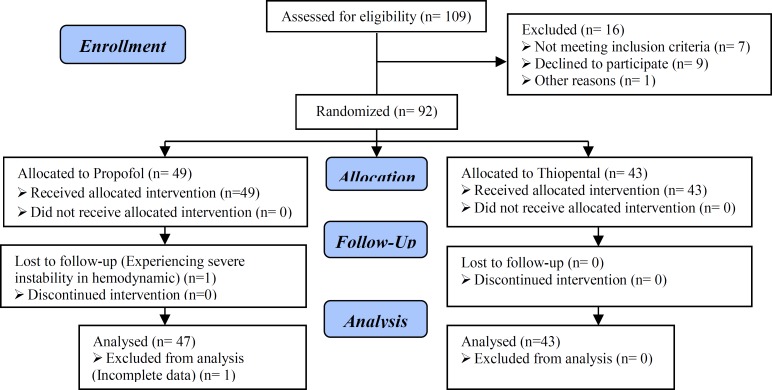
Consort flow diagram

Also all the patients who exposed to an unintended clinical problem during the anesthesia excluded from the study Informed consent form was conducted for all samples. Ethic Committee of Shahid Sadoughi University of Medical Sciences confirmed the plan of the study (Reference number: 3763). Consort flow diagram of this study show the population and exclusion step by step (Figure 1). 

**Data Collection Procedure**

The thiopental (Nani pharmaceutical ltd. India) and Propofol (Dangkook Pharmacology ltd. South Korea) groups were respectively induced with 5mg/kg of thiopental Sodium and 2.5 mg/kg of Propofol. Before the induction process, all the participants underwent a pre-oxygenation and then, received 1 mg/kg of midazolam and 2 mg/kg of fentanyl. In cases where a longer consciousness was needed, again Propofol 40 mg for the Propofol group and thiopental 100 mg for the thiopental group were used while no cases needed additive drugs. 

Systolic and diastolic blood pressures and pulse rates of the participants were measured immediately after the induction and then at five-minute intervals (10, 15, 20 minutes after start) during the experiment. Mature MII oocytes were selected to be included in the ICSI process. After 16-18 post-oocyte microinjections, all oocytes were microscopically observed for signs of fertilization. Fertilization was confirmed when two pronuclei were present within the ooplasm. The rate of fertilization was calculated as the percentage of the fertilized oocytes per MII oocytes. Oocyte quality and level of βHCG were evaluated in two groups.

Exactly 24 hours after fertilization, cleaved embryos were assessed and graded according to the degree of fragmentation and size of blastomeres. These were categorized into four groups: A (score 18-20), B (score 16-17), C (score 14-15) and D (score 12-13) (10). In general, grade D embryos were discarded. Also, Transvaginal oocyte retrieval was done using a 17-gauge needle using ultrasound guidance. When the needle was introduced into a follicle, suction was applied to 90-100 mm Hg until the follicle was emptied. This process was performed for each ultrasonically visible follicle larger than 12 mm in diameter.

**Statistical analysis**

The data from the samples were analyzed using ANOVA, Chi-square, Fishers Exact test, and Mann-Whitney U-test. The analytical software employed in the study was SPSS version 20. The significance level for the study was considered to be p>0.05. 

## Results

Mean of age in Propofol group was 30.23±4.42 versus 28.72±5.19 years in Thiopental sodiumgroup (p=0.26). While 12 (26.7%) of the participants in Thiopental sodium group had vomiting, it was not a case in the Propofol group. 49 cases entered experiment group (receiving Propofol), one case experienced severe unstable hemodynamic and excluded from the study. Also another case excluded due to incomplete data in analyzing and patients (n=43) in thiopental group had no exclusion, 4 (31.1%) of the participants in the thiopental group were reported to have nausea while no case in the Propofol group was observed. 

The results of the analysis indicated that vomiting (p=0.001) and nausea (0.000) in the Propofol group were significantly less than the thiopental group. Moreover, oocyte quality (p=0.23), fertility success (p=0.09), cleavage outcome (p=0.12), and βHCG results were not significantly different (p=0.33). Mean of the time of rapid response to the acoustic stimulation in Propofol was 3.25±1.58 seconds while this time in Thiopental sodium group recorded as 6.35±1.99 seconds, Mann Whitney U test showed a significant difference between two groups (p=0.001). Data of vital sign during anesthesia (Table I) and recovery (Table II) are shown in figurs 2-4.

**Table I:** Vital signs measurement during anesthesia

**Vital sign**	**Group**	**Time during anesthesia (min)**				
		**0**	**5**	**10**	**15**	**20**
SBP						
	Propofol	107.15 ± 11.86	106.76 ± 14.62	103.26 ± 12.16	108.57 ± 10.83	109.34 ± 9.37
	Thiopental sodium	110 ± 13.36	108.4 ± 12.47	109.77 ± 12.09	109.09 ± 14.19	110.68 ± 11.98
	p-value*	0.12	0.55	0.09	0.86	0.95
DBP						
	Propofol	68.26 ± 11.04	66.15 ± 14.71	67.11 ± 16.19	70.03 ± 15.25	69.69 ± 14.39
	Thiopental sodium	70 ± 14.55	68.86 ± 14.81	71.36 ± 14.81	71.59 ± 13.12	72.95 ± 13.68
	p-value*	0.36	0.45	0.23	0.85	0.33
PR						
	Propofol	93.77 ± 15.22	87.4 ± 14.51	82.11 ± 13.34	87.7 ± 1089	78.88 ± 1099
	Thiopental sodium	94.95 ± 12.41	89.72 ± 11.14	87.9 ± 8.26	86.68 ± 10.82	84.09 ± 10.99
	p-value*	0.66	0.563	0.07	0.12	0.06

SBP: Systolic blood pressure

DBP: diastolic blood pressure

PR: pulse rate/min

* Mann Whitney U Test

**Table II:** Vital signs measurement during recovery

**Vital sign**	**Group**	**Time during recovery (min)**				
		**0**	**5**	**10**	**15**	**20**
SBP						
	Propofol	108.62 ± 11.02	107.4 ± 10.31	110.55 ± 11.46	111.85 ± 10.66	112.77 ± 9.74
	Thiopental sodium	111.81 ± 11.18	112.27 ± 9.96	111.36 ± 8.88	112.04 ± 8.95	113.88 ± 1034
	p-value*	0.21	0.33	0.08	0.07	0.06
DBP						
	Propofol	70.37 ± 12.55	73.39 ± 9.68	71.11 ± 12.18	71.48 ± 11.99	71.85 ± 11.44
	Thiopental sodium	72.5 ± 12.07	73.4 ± 11.68	72.95 v 11.19	73.18 ± 11.29	74.09 ± 11.81
	p-value*	0.33	0.99	0.85	0.36	0.56
PR						
	Propofol	85.92 ± 10.92	83.58 ± 8.68	84.8 ± 8.68	84.73 ± 8.56	84.46 ± 7.56
	Thiopental sodium	84.81 ± 10.21	83.59 ± 9.69	82.72 ± 10.92	83.31 ± 10.31	83.4 ± 8.36
	p-value*	0.86	0.99	0.51	0.23	0.32
RR						
	Propofol	15.77 ± 3.57	15 ± 2.46	14.29 ± 2.03	14.01 ± 2.96	14.22
	Thiopental sodium	15.59 ± 2.97	15.09 ± 2.77	15.27 ± 2.33	15.04 ± 2.01	15.09 ± 2.13
	p-value*	0.92	0.97	0.85	0.34	0.175

SBP: Systolic blood pressure

DBP: diastolic blood pressure

PR: pulse rate/min

RR: respiratory rate/ min

* Mann Whitney U Test

**Figure 2:**
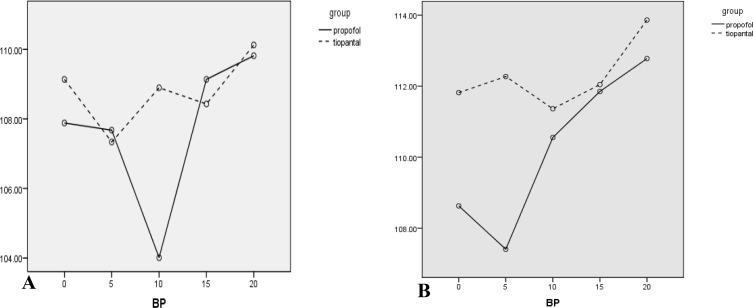
Systolic blood pressure means (mmHg) of the participants during different anesthesia (A) and recovery (B) periods are shown. During anesthesia (A) an obvious difference is seen in minute 10, Also in recovery (B) during 0 and 5 minutes difference appears to be important but no intervals in this figure during separated times were significantly different

**Figure 3:**
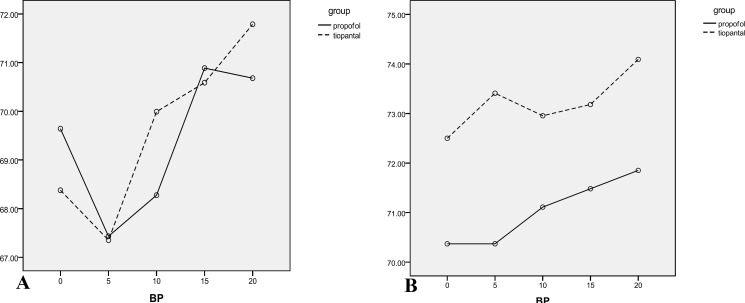
Diastolic blood pressure means (mmHg) of the participants during different anesthesia (A) and recovery (B) periods are shown. No significant difference during different intervals in separated times is seen

**Figure 4:**
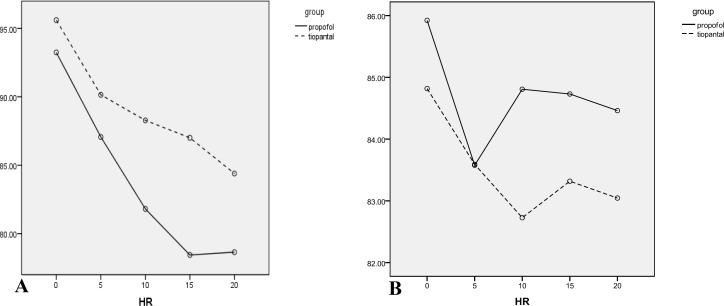
Pulse rate means (mmHg) of the participants during different anesthesia (A) and recovery (B) periods are shown. No significant difference during different intervals in separated times is seen

## Discussion

Since the first time anesthetics were observed in the follicular fluid, controversial theories have been proposed for the deleterious effects of anesthetics with a focus on their probable side effects (2, 3). As an anesthetic, Propofol has enjoyed a continuous popularity. The results of a study conducted in Taiwan showed no statistically significant difference between thiopental and Propofol in terms of fertility, cleavage, pregnancy, fertility, and abortion rate. Particularly, less vomiting and nausea was reported for thiopental in the study (11). Also, Pierce *et al* investigated the pregnancy rate after GIFT (Gamete Intra-fallopian Transfer) and found no significant difference between thiopental and Propofol (12). 

The findings of the present study are similar to those of the above-mentioned studies since no significant difference was found between the two groups in terms of oocyte quality, pregnancy and cleavage outcomes, and βHCG on the 15^th^ day after embryo implantation. Of course, among the studies referred to above none had investigated the βHCG on the 15^th^ day after embryo implantation. In all similar studies Propofol is preferable to thiopental. Therefore, in a number of other studies Propofol has been compared with other drugs during the ART process. In a study done in 2008, the effect of local anesthesia on ART was explored by injecting Remi fentanyl. The findings of the study suggested that Remi fentanyl had no effect on the retrieved oocyte (13). 

The results of a study conducted in the USA indicated that Propofol and Isoflurane had no impact on pregnancy rate in GIFT (14, 15). The findings of another study in the USA did not report a huge difference in the pregnancy and fertility rates of the 117 women anaesthetized by Propofol in comparison with those who did not received the drug during oocyte retrieval (16). In other hand, Propofol provides rapid onset and offset with context-sensitive decrement times of approximately 10 minutes when infused for less than 3 hours and less than 40 minutes when infused for up to 8 hours. 

Its mechanism of action is thought to be potentiation of γ-amino butyric acid (GABA)-induced chloride currents (17). At therapeutic doses, Propofol produces a moderate depressant effect on ventilation. It causes a dose-dependent decrease in blood pressure primarily through a decrease in cardiac output and systemic vascular resistance. A unique action of Propofol is its antiemetic effect, which remains present at concentrations less than those producing sedation (18). In many other surgeries the effects of Propofol and thiopental have been compared (19). This is due to the fact that thiopental has been one of the most widely-used anesthetics and with the introduction of Propofol, it is giving way to Propofol. It should be noted that the effects of Propofol have been compared with other drugs, too.

## Conclusion

In the light of the findings of the present study and other similar studies, it can be argued that there is no significant difference between thiopental sodium and Propofol in terms of pregnancy outcomes. Also, there is no evidence suggesting that the two drugs can influence oocyte quality and fertility rate negatively. However, Propofol is followed by less vomiting and nausea. Moreover, Propofol has a better performance in controlling the pulse rate of patients. In addition, it guarantees a faster recovery, and makes it possible for patients to get their normal breathing back quicker. Therefore, it is recommended that Propofol be used as the anesthetic option for surgeries required during ART.
